# Gaming disorder and problematic caffeine consumption in adolescents: a narrative review and public health framework

**DOI:** 10.3389/fpsyg.2025.1690304

**Published:** 2025-10-09

**Authors:** Jennifer J. Park, Yihong Zhao, Marc N. Potenza

**Affiliations:** ^1^Department of Psychiatry, Yale University School of Medicine, New Haven, CT, United States; ^2^Columbia University School of Nursing, New York, NY, United States; ^3^Child Study Center, Yale University School of Medicine, New Haven, CT, United States; ^4^Department of Neuroscience, Yale University School of Medicine, New Haven, CT, United States; ^5^Connecticut Council on Problem Gambling, Wethersfield, CT, United States; ^6^Connecticut Mental Health Center, New Haven, CT, United States; ^7^Wu Tsai Institute, Yale University, New Haven, CT, United States

**Keywords:** gaming disorder, problematic caffeine consumption, adolescents, review, public health, addictive behaviors, internet addiction, caffeine

## Abstract

Caffeinated beverages, particularly energy drinks, appear to be frequently consumed by adolescents during gaming sessions. This may be in part due to the normalization of caffeine use within gaming culture, where energy drinks are marketed as performance-enhancing tools and symbols of the “gamer” identity. Given evidence of the relationships between gaming disorder (GD) and problematic caffeine consumption (PCC), a narrative review was conducted to synthesize the current literature on GD/gaming and PCC/caffeine use in adolescents. Eight key topics emerged from the literature: (1) the associations between GD/gaming and PCC/caffeine use; (2) the need for increased regulation of caffeine sales and marketing in gaming contexts; (3) parental and peer influences on caffeine use; (4) health and marketing literacy related to caffeinated products; (5) sex-specific patterns in gaming-related caffeine consumption; (6) overlapping neurocognitive mechanisms underlying GD and PCC; (7) self- or peer-enacted strategies for reducing caffeine intake; and (8) shared negative consequences of GD and PCC. These findings reveal multiple interacting influences that may reinforce both behaviors and have been used to propose a public health framework to inform future research, intervention, and policy to promote and protect the health of adolescents who consume caffeine and game.

## 1 Introduction

Approximately 33%−80% of individuals report concurrent caffeine consumption during gaming sessions, positioning caffeine as the most commonly used substance in gaming contexts (Carey et al., 2022; Ip et al., 2021; Matias et al., 2023; Ream et al., 2011; Škarupová et al., 2018). The rate and popularity of caffeine consumption is particularly concerning in adolescents, who not only represent the primary demographic for the consumption of energy drinks and caffeinated sodas, even beyond the context of gaming (Drewnowski and Rehm, 2016), but also exhibit gaming disorder (GD) at a high prevalence (Kim H. S. et al., 2022). According to the eleventh revision of the International Classification of Diseases (ICD-11), GD is characterized by persistent or recurrent gaming behavior marked by impaired control over gaming, prioritization of gaming over other activities and interests, and continued or escalated engagement despite significant negative consequences (World Health Organization, 2019b). The ICD-11 also recognizes problematic caffeine consumption (PCC) in the category of “disorders due to use of caffeine” (6C48), including “harmful pattern of use of caffeine” (6C48.1) characterized by continuous, recurrent, or sporadic caffeine consumption leading to significant negative consequences to physical or mental health (World Health Organization, 2019a).

Most patterns of habitual caffeine consumption among adolescents may reasonably be classified as PCC, considering recommendations from several public health agencies to limit adolescent caffeine intake to no more than 100 mg per day and to avoid energy drinks entirely before adulthood (American Academy of Child and Adolescent Psychiatry, 2020; American Academy of Pediatrics, 2023; Lopez Frias, 2021). However, youth-targeted marketing of caffeinated beverages, particularly energy drinks, appears to directly undermine these public health guidelines, with advertisements embedded in gaming platforms and events where regulatory oversight may be limited and adolescents may be especially impressionable (Ayoub et al., 2023; Lopez Frias, 2021; Roscoe et al., 2023; Yang et al., 2022). This incongruence between public health recommendations and commercial practices raises concerns about the normalization of caffeine consumption and associated harms (e.g., sleep concerns) among adolescents, particularly those who game.

Despite the ubiquitous presence of caffeine in gaming environments, scholarly attention to the intersection of PCC/caffeine use and GD/gaming among adolescents appears limited. Much of the literature on substance-use-related gaming has focused on alcohol, tobacco, and cannabis (Burleigh et al., 2019), potentially overlooking the unique position of caffeine as both a socially accepted stimulant and a product frequently marketed directly to youth who game. Unlike other substances, energy drink brands frequently leverage esports sponsorships and influencer endorsements to position caffeine not merely as a potential performance enhancer, but as a vehicle for identity formation (“gamer” identity), social belonging, and achievement within gaming communities (Edwards et al., 2022; Roscoe et al., 2023; Slater, 2015). Thus, there is a need for a better understanding of caffeine use among adolescents who game.

The present narrative review aimed to synthesize the existing literature on GD/gaming and PCC/caffeine use in adolescents. Although energy drinks appear to be the most closely associated with gaming engagement, marketing, and culture (Andrade et al., 2023; Lopez Frias, 2021; Yang et al., 2022), other caffeinated drinks (e.g., coffee and caffeinated sodas) also show relevant gaming-related associations that should be explored further (Edwards et al., 2022; Klose and Truong, 2022). Accordingly, this review considers all forms of caffeinated beverages as well as unspecified or general caffeine use among adolescents. This review summarizes the relationship between GD/gaming and PCC/caffeine use, caffeine sales/marketing regulations in the context of gaming, parental and peer influences on caffeine consumption, and the influence of caffeine-related health and marketing literacy. Additionally, the review discusses sex-specific differences in gaming-related caffeine consumption, neurocognitive mechanisms related to GD and PCC, self-enacted or peer-based strategies for caffeine reduction/abstinence, gaming-related motivations for caffeine use, and overlapping negative consequences for GD and PCC. Across these topics, limitations and implications are considered. We also propose a public health framework to summarize the findings of this narrative review and inform future research, policy, and interventions for behavioral change in adolescents who engage in both gaming and caffeine use.

## 2 Methods

A narrative review was conducted by searching electronic databases, including PubMed, Web of Science, and PsycINFO, between May and August 2025. The search strategy used various keywords related to GD/gaming and PCC/caffeine use, such as “problematic gaming” and “caffeine use disorder,” in combination with topic-specific terms (e.g., “adolescents,” “regulation,” “marketing,” “motivations”). Articles were screened for relevance, and findings in relevant literature were deductively categorized and synthesized under thematic subheadings. Only articles published in English were included. Gray literature (e.g., reports and theses) was also excluded. For the purposes of this review, adolescents were defined as individuals aged 10–19 years, in accordance with the World Health Organization's classification (World Health Organization, 2025). Eighty-nine articles were included in this narrative review, representing 575,389 participants.

## 3 Results

### 3.1 The relationship between GD/gaming and PCC/caffeine use

Despite caffeine being the most commonly consumed substance during gaming sessions, relatively few studies have investigated the relationship between GD and PCC in youth-only samples. However, studies have examined and reported a positive relationship between GD and PCC in mixed samples that include both youth and adults (Porter et al., 2010; Ream et al., 2013). In an international survey of 1,945 adolescents and adults who engaged in gaming (54% aged 14–19 years; 93% male), a significantly higher proportion of individuals with GD reported “excessive” caffeine consumption compared to individuals without GD (Porter et al., 2010). Similarly, computer-assisted personal interviews using life-history calendars with 702 youth and young adults involved in gaming in the US (mean age = 21.2, SD = 3.1; 66% male) found that PCC statistically predicted GD (Ream et al., 2013).

Regarding caffeine use (not specified as problematic) and GD, studies similarly remain scarce. In a study of 2,749 young adolescents engaged in gaming in the US (61% male), greater GD severity at the two-year follow-up (mean age = 11.9) was significantly associated with increased caffeine use at the third-year follow-up (Park et al., 2025). A survey study with 2,613 seventh-grade students in Taiwan (47% male) reported a significant link between the consumption of energy drinks specifically and GD (Yang et al., 2022). Another survey with 3,952 Czech adolescents and adults who played massive multiplayer online (MMO) games (55% aged 11–21 years; 92% male) found higher GD scores in individuals who consumed caffeine compared to those who did not (Škarupová et al., 2018). However, one US survey of 4,028 high school students (age range = 14–18; 76% male) found contrasting results, with problematic gaming being associated with lower caffeine consumption (Desai et al., 2010), with these data having been collected more than 20 years ago, in a different gaming environment than exists presently. Games were less immersive, online multiplayer (i.e., potentially one of the most addictive game types) access was more limited, and esports and streaming were not yet mainstream (Ivory, 2015; Kuss et al., 2012). Additionally, gaming-related marketing of energy drinks and other caffeinated products was far less pervasive than today (Kerr et al., 2025; Smith et al., 2014), meaning adolescents may have been less frequently exposed to promotional cues linking gaming with caffeine use. Such historical differences in both the gaming environment and the caffeinated beverage market may have contributed to the different findings across studies.

Some studies (Bradbury et al., 2019; Larson et al., 2014; Ream et al., 2013; Škarupová et al., 2018) have found positive associations between caffeine use and gaming (both not specified as problematic). A survey of 2,793 middle- and high-school students in the US (mean age = 14.4, SD = 2.0; 47% male) found that adolescent males and females who regularly consumed energy drinks spent more time gaming per week than those with lower energy-drink consumption (Larson et al., 2014). Another US study of young adolescents engaged in gaming (described previously as *N* = 2,749; 61% male) found a significant link between average daily gaming duration at the second-year follow-up (mean age = 11.9) and caffeine use at the third-year follow-up (Park et al., 2025). A survey of individuals playing MMOs (described previously as *N* = 3,952; 55% aged 11–21 years; 92% male) found that individuals who used caffeine gamed 3.8 more hours per week on average compared to those who did not consume caffeine (Škarupová et al., 2018). This study also found that the former group reported higher gaming engagement scores, which authors defined as increased engagement that does not meet their specified criteria for addiction. Computer-assisted personal interviews with individuals involved in gaming (described previously as *N* = 702; mean age = 21.2, SD = 3.1; 66% male) identified caffeine use as a reliable statistically significant predictor of gaming (Ream et al., 2013). Lastly, a study analyzing data from 32,418 students in grades 8 and 10 in the US found that (i) caffeine consumption was significantly higher by 2.78 mg per hour of gaming, (ii) gaming was associated with an estimated intake of 4.4 additional mg of caffeine per day, and (iii) the odds of exceeding caffeine recommendations (from the World Health Organization) were significantly higher by 4% per hour of gaming (Bradbury et al., 2019).

### 3.2 Regulation of caffeine sales/marketing and links to gaming culture

The sale of caffeinated beverages remains legal for minors (typically under the age of 18 or 21 years) in most countries. However, the unique risk profile of energy drinks, which often contain high doses of caffeine alongside other stimulants and sugar and involve youth-targeted marketing, has recently prompted regulations in several countries. A systematic review identified that energy-drink-related sales bans, bans in schools, and advertising and marketing bans (*n* = 33, 23, 14 countries, respectively) were mostly adopted in Europe, North and South America, and Oceania, with many countries implementing multiple strategies simultaneously (Rostami et al., 2024). Since that review, further regulatory measures have been enacted, reflecting international recognition of energy drinks as a public health concern for adolescents. For example, Poland introduced a nationwide ban in 2024 on the sale of energy drinks containing caffeine and taurine to minors under 18 (Mularczyk-Tomczewska et al., 2025). Focus groups with young adolescents have shown general support for structural regulations on the sale and marketing of energy drinks to minors (Visram et al., 2017). However, two focus group studies highlighted limitations of school-based bans, such as the formation of “black markets” within schools, where students purchase prohibited products such as energy drinks from peers (Fletcher et al., 2014; Visram et al., 2017). These transactions are often coordinated through online messaging platforms, highlighting the adaptability of youth networks in circumventing restrictions (Fletcher et al., 2014).

The need to regulate marketing approaches appears to be a topic of particular concern, given the symbiotic relationship between gaming culture and energy drink consumption (Roscoe et al., 2023; Yang et al., 2022). Despite inconclusive evidence around the effectiveness of caffeine as a performance-enhancing drug for gaming (Lopez Frias, 2021; Ribeiro and Poínhos, 2023; Thomas et al., 2019), energy drinks are frequently marketed as tools for enhancing gaming performance, concentration, or stamina. One article discussing the ethics of marketing energy drinks to individuals who game described youth-targeted messages, such as “victory in a can (that) tastes like berry-flavored win sauce” and “domination in a can (that) tastes like losers' tears” (Lopez Frias, 2021). Such claims may resonate with adolescents forming “gamer” identities and align with commonly reported gaming motivations, such as competition, identity expression, and social status (Neys et al., 2014; Razum and Huić, 2024; Roscoe et al., 2023). Youth-oriented marketing tactics target both competitive and recreational gaming culture by branding energy drinks as “game fuel,” sponsoring esports tournaments, and working with high-profile streamers (i.e., individuals broadcasting live gaming who are typically viewed as online celebrities/influencers), with some streamers having their own flavor collaborations (Evans et al., 2024). Energy drink brands also collaborate with specific video games to deliver in-game pop-up advertisements or provide game-specific promotions to consumers. For instance, focus groups with young adolescents highlighted that certain energy drinks feature codes printed under the ring-pull tab, which offer rewards that would directly influence gameplay (Visram et al., 2017). Rewards such as experience point (“XP”) boosts and exclusive skins for weapons and avatars offer status-enhancing and performance-related benefits, which may be particularly attractive to adolescents.

The reach of marketing campaigns appears to be extensive. Exposure to energy drink advertisements online was reported by 44%−46% of youth in Canada (Hammond and Reid, 2018; Wiggers et al., 2019), 75% in Taiwan (Yang et al., 2022), and 82% in Australia (Parnell et al., 2023). Adolescents in Australia also specifically reported seeing energy-drink advertisements on social media (62%) and video games (34%) (Parnell et al., 2023). The widespread reach of marketing may be attributable to the use of engagement-boosting strategies on social media (e.g., the use of hashtags and prompts to like, comment, and share) and the incorporation of adolescent-appealing themes such as gaming. One study reported that 93% of Instagram/Facebook posts and 73% of Twitter posts by energy drink companies employed the former and latter strategies, respectively (Ayoub et al., 2023). Individuals who game may have higher exposure to the marketing of energy drinks given the concentration of advertisements on gaming-related platforms and during events. Energy drinks were found to be the most featured food type in a study examining food and non-alcoholic beverage marketing via Twitch (i.e., a live-streaming platform for games with a large adolescent following) streamers of the game “Fortnite” (Evans et al., 2024). Though far less frequently marketed than energy drinks (70.4% of all branded marketing cues), caffeinated soda (5.6%) and coffee (0.9%) were also marketed by Fortnite streamers popular with adolescents on Twitch. Furthermore, a study of youth and adults found that in-game advertising for caffeinated soda beverages (during a collaboration between a game and a soda brand) increased intended duration and frequency of gaming, efforts to win rewards during the collaboration event, and intentions to make in-game purchases (Klose and Truong, 2022). More widespread regulations for energy-drink, coffee, and caffeinated-soda marketing may be warranted, given evidence linking greater advertising exposure to higher levels of consumption in young adolescents and increased consumption being linked to GD (Yang et al., 2022). Successful implementation of advertising regulations may require cooperation across various sectors, including energy-drink, caffeinated-soda, coffee, and game companies. For instance, despite the presence of Canada's Energy Drinks Marketing Code that regulates marketing to children, 42% of individuals aged 12–14 years still perceived these energy drink advertisements as targeting people their age or younger (Hammond and Reid, 2018).

### 3.3 Parental and peer influences on caffeine consumption

Given that energy drinks are predominantly marketed online (e.g., in video games and social media accounts promoting video games), parents may have limited experience and comfort with monitoring and limiting the influence on children's attitudes and behaviors around caffeine consumption (Buckingham, 2005; Lopez Frias, 2021). In the advertising industry, children are often portrayed as media-savvy, discerning consumers. Such assumptions may obscure the need for safeguards and further diminish parents' capacity to protect children from marketing tactics (Buckingham, 2005), which may be particularly prevalent and persuasive in gaming culture (Lopez Frias, 2021). However, parents, caregivers, and other influential adults often contribute significantly to adolescents' access to energy drinks, either through facilitation or restriction (Andrade et al., 2023; Ludden et al., 2017; Visram et al., 2017). In focus groups with young adolescents, some youth reported that family members were ambivalent about their consumption of energy drinks, occasionally offering them as rewards or post-exercise hydration (Visram et al., 2017). Others described adults who actively discouraged use, citing health and behavioral concerns. Sports coaches were also identified as important role models. For example, one adolescent recounted his coach advising, “Don't drink them before football, just bring some water,” emphasizing the coach's concern for players' wellbeing: “The coach just cares for you, and he wants to look out for you. And he doesn't want your heart full of junk” (Visram et al., 2017). This highlights the potential value of similarly trusted figures in online spaces, such as gaming streamers and esports players, who could play a proactive role in discouraging energy drink consumption among young adolescents.

Caffeine consumption among adolescents who game also appears to be shaped by peer influences, often serving as a means of social bonding within gaming contexts (Visram et al., 2017). Marketing strategies link energy drink consumption to gaming culture, which may encourage adolescents to use these products to project a gamer identity and signal affiliation with gaming communities. Specifically, young people may consume energy drinks to imitate gaming influencers, fit in with peers, or feel a sense of group camaraderie through shared social rituals (Lopez Frias, 2021; Roscoe et al., 2023). For example, focus groups with young adolescents found that energy drinks were integrated into social gaming routines (e.g., sleepovers involving gaming sessions) where both purchasing and consuming energy drinks became part of the collective experience (Visram et al., 2017). Similarly, in a study involving both older adolescents and adults who game, participants described using energy drinks with peers to generate “hype and anticipation” before intense sessions of gaming (Roscoe et al., 2023). Peer dynamics also played a role in shaping preferences for energy drink brands, further emphasizing the socially embedded nature of caffeine consumption within gaming environments (Roscoe et al., 2023).

Notably, young adolescents did not frame their energy drink consumption solely in terms of peer pressure or a desire to fit in Visram et al. (2017). Instead, they offered nuanced reflections on the influence of targeted marketing practices, such as participants demonstrating their understanding of certain energy drinks having links with particular games and the use of specific strategies to attract young, male consumers. Participants expressed support for policy-level or school-based interventions, such as restricting access through increasing pricing, placing energy drinks in store aisles that are inaccessible to adolescents, or school-based lessons to raise awareness of the risks of caffeine consumption during young adolescence, which may prompt voluntary behavioral change (Visram et al., 2017).

### 3.4 Caffeine-related health and marketing literacy

Greater online marketing literacy around energy drinks advertised in gaming contexts (e.g., “the live broadcaster of the game mentioned in the live broadcast that he relies on a certain brand of energy drinks to refresh himself, which are marketing techniques”) has been associated with lower energy drink consumption in adolescents (Yang et al., 2022). Although many adolescents appear to be familiar with energy drink brands and associate them with high caffeine content, understanding of what constitutes “high” caffeine levels remains limited (Visram et al., 2017). In a study of seventh- and eighth-grade students, more than half could not accurately identify which beverages had the most caffeine, and nearly 29% were unaware their favorite drinks contained caffeine (Thakre et al., 2015). Some young adolescents also expressed confusion about how exactly energy drinks affected health (Visram et al., 2016). Limited awareness may also persist into older adolescence, with some adolescents perceiving energy drinks as similar to sports drinks and generally safe (Kraak et al., 2020). This misconception may be in part due to advertisements of new energy drinks emphasizing supposedly health-conscious features, such as zero calories and no added sugars, which may obscure potential risks associated with high caffeine content (Kraak et al., 2020). Adolescents have reported encountering such messaging via energy-drink advertisements within games, with just some expressing skepticism toward the positive health claims (Visram et al., 2016).

There is a need for comprehensive public health programs, given the general lack of health literacy regarding the understanding of caffeine content in beverages and the limited awareness of health-related risks among adolescents. These may include prevention efforts aimed at limiting youth access to high-caffeine products, harm-reduction approaches that promote safer consumption practices, and health-promotion initiatives that improve caffeine-related knowledge and decision-making skills. Considering the potential impact of increasing caffeine-related health literacy among adolescents who game or have GD, it may also be important to consider other social determinants of health that may influence caffeine consumption and PCC in this population (Kaldenbach et al., 2021).

### 3.5 Sex-specific differences

Adolescent males who regularly consumed energy drinks (defined as consuming at least one energy drink per week) were found to spend approximately 4.5 more hours gaming per week compared to adolescent males with lower consumption (Larson et al., 2014). In contrast, adolescent females in this category spent around 2.5 additional hours gaming per week compared to their peers with lower consumption (Larson et al., 2014). Regarding the consumption of energy drinks more broadly, research suggests that consumption patterns of energy drinks and associated concerns may be more prevalent among boys than girls (Andrade et al., 2023; Carneiro et al., 2025; Ludden et al., 2017; Svensson et al., 2021; Temple, 2019; Visram et al., 2017). Thus, public health approaches and interventions may benefit from tailored sex-specific messaging to effectively engage different groups (Temple, 2019). Beyond consumption behaviors, boys reported being more influenced by energy drink advertising. On the other hand, girls demonstrated greater online marketing literacy (Yang et al., 2022), which may act as a protective factor against the influence of energy-drink marketing within gaming contexts.

### 3.6 Neurocognitive mechanisms

Both GD and PCC may engage overlapping neurocognitive processes commonly seen in addictions. Each may reinforce behavior through both positive rewards (e.g., excitement from gaming or energy boost from caffeine) and negative reinforcement (e.g., relief from stress or fatigue) (Brand et al., 2025a, 2016; Griffiths and Woodson, 1988). Neuroimaging studies have reported altered reward systems among individuals with GD and other internet addictions, including lower dopamine D2 receptor availability in the dorsal striatum (Kim et al., 2011), greater cue-related activation across ventral and dorsal striatal regions, and stronger left ventral striatal activation being correlated with lower craving (Liu et al., 2017). Similarly, the pharmacological action of caffeine (adenosine blockade) has been reported to facilitate dopaminergic activity in the nucleus accumbens in animal studies, suggesting that caffeine may influence mesolimbic reward pathways that may underlie reinforcing effects (Solinas et al., 2002).

Impulsivity represents a transdiagnostic neurocognitive mechanism that reflects a tendency to act quickly in response to stimuli (e.g., gaming-related or caffeine-related) without adequate reflection or assessment of possible consequences (Moeller et al., 2001). Individuals with GD have displayed higher impulsivity and poorer response inhibition (Chen et al., 2021). Although there have been no studies examining the impact of increased caffeine intake on impulsivity related to GD, one study reported that higher caffeine consumption was significantly associated with greater personality-related impulsiveness and impulsive decision-making among individuals with gambling disorder (Grant and Chamberlain, 2018). With repeated reinforcement, both gaming and caffeine consumption may shift toward more habit-like, compulsive patterns of use. For example, compulsivity in behavioral addictions has been described as involving goals that evolve from reward or relief-seeking to more rigid, habitual, and seemingly automatic patterns of engagement (Brand et al., 2025b).

### 3.7 Peer-based or self-enacted strategies for caffeine reduction/abstinence

Although no studies have specifically examined strategies for caffeine reduction among adolescents who game, research involving adolescents more broadly (including those who may engage in gaming) provides insight into potential avenues for intervention. Focus groups with young adolescents have reported instances where peer groups collectively chose to abstain from or reduce consumption of energy drinks (Visram et al., 2017), underscoring the potential for peer-led interventions to be studied in the future. This study also specifically discussed the potential of training adolescent “champions” to disseminate information and advise peers regarding consumption of energy drinks (Visram et al., 2017).

In another focus group of high school students, participants were not directly asked about reasons for avoiding caffeine, but several adolescents voluntarily cited disliking the taste of energy drinks, soda, or coffee, and experiencing negative physiological effects, including jitteriness, poor sleep, headaches, and stomach pain (Ludden et al., 2017). Some students also reported avoiding caffeine during school hours due to concerns about its potential impact on academic performance or disliking sugary caffeinated beverages due to long-term negative consequences to health (Ludden et al., 2017). These findings suggest that adolescents are capable of articulating clear, self-generated reasons for behavioral change. In the context of gaming, where caffeine use is often normalized or even encouraged, building on adolescents' existing motivations and fostering peer-supported efforts (or other low-intensity interventions and strategies) may be particularly effective in reducing excessive consumption. For example, a qualitative study on the lived experience of caffeine reduction identified that the most commonly used behavior change techniques were self-managed, including substance substitution, information seeking, and avoidance strategies (Rodda et al., 2020). This aligns with survey findings indicating that the most frequently endorsed help-seeking option for PCC was searching for information online, followed by the use of self-directed resources, while formal services (e.g., counseling or helpline services) were far less preferred (Booth et al., 2020).

### 3.8 Gaming-related motivations

When asked about reasons for consuming caffeine during gaming sessions, adolescents and adults most commonly discussed the following game-related motivations: sleep avoidance, improved concentration, increased enjoyment, management of tension, and greater courage (Škarupová et al., 2018). Caffeine use during gaming appears to be particularly common or popular among individuals who play first-/third-person shooter, multiplayer online battle arena, role-playing, racing, sports, and fighting games (Roscoe et al., 2023; Škarupová et al., 2018). For example, 76.4% of participants who played first-/third-person shooter games reported consuming caffeine while gaming, compared to lower percentages for other substances; e.g., 45.5% reported alcohol use, 24.5% tobacco use, and 15.1% cannabis or resin use (Škarupová et al., 2018). These game genres are typically more fast-paced, competitive, and cognitively or physically demanding than strategy and social simulation games, which aligns with commonly reported reasons for concurrent gaming and caffeine use (Škarupová et al., 2018). Considering studies that have reported associations between personality features and specific game genres (e.g., shooting games have been linked to lower self-esteem in individuals who game compared to individuals who do not) (Kim D. et al., 2022; López-Fernández et al., 2021), further research is warranted to examine how psychological characteristics may influence patterns of caffeine use among adolescents who game.

### 3.9 Overlapping negative consequences

There are several overlapping negative consequences of GD and PCC in adolescents, with the most common appearing to be sleep concerns, such as reduced sleep duration, poor sleep quality, and increased sleep problems (Bonnar and Gradisar, 2015; Carneiro et al., 2025; Cusick et al., 2020; Kristensen et al., 2021; Watson et al., 2017). A recent study of 2,749 young adolescents who game also found that caffeine consumption partially mediated the relationship between gaming duration/disorder and sleep duration/difficulties (Park et al., 2025). The dominant component of the mediated effect was the consumption of energy drinks (in the relationship between gaming duration and sleep difficulties) or the combination of energy drinks and caffeinated soda (in the relationship between GD and sleep duration) (Park et al., 2025). Depression and anxiety have also been frequently associated with both GD and increased caffeine intake in adolescents (Andrade et al., 2023; Cho et al., 2024; Fontelles et al., 2025; Jin et al., 2016; Luebbe, 2011; O'Neill et al., 2016; Richards and Smith, 2015; Uddin et al., 2017).

Some studies have discussed caffeine as a potential gateway drug to other substances (Galimov et al., 2019, 2020; Yang et al., 2022), and several longitudinal studies have reported the association between caffeine consumption in early adolescence and increased likelihood of alcohol use in adulthood (Arria et al., 2017; Brunborg et al., 2022; Choi et al., 2016; Galimov et al., 2020; Kristjansson et al., 2022; Marmorstein, 2019; Miyake and Marmorstein, 2015; Patrick and Maggs, 2014) as well as early onset of alcohol use (Kristjansson et al., 2022). Furthermore, adolescents reporting both consumption of energy drinks and daytime fatigue and/or initial insomnia have been found to exhibit particularly high likelihoods of alcohol use (Marmorstein, 2017). Similarly, gaming disorder has been linked to early substance use (Mihara and Higuchi, 2017) and increased/problematic use of a range of substances, including alcohol, cannabis, and nicotine in adolescents (Di Carlo et al., 2023; Van Rooij et al., 2014). Both GD and caffeine use have been associated with polysubstance use (Horváth et al., 2022; Yudko and McNiece, 2014), with emerging evidence also suggesting increased polysubstance use involving caffeine among youth who game (Di Carlo et al., 2023). Of particular concern is the concurrent use of caffeinated energy drinks and alcohol, typically called “AmED” (Alcohol Mixed with Energy Drinks) or alcohol-energy drinks (Andrade et al., 2023; Mallett et al., 2015; Patrick et al., 2018; Pollard et al., 2015). The ability of caffeine to reduce perceived alcohol intoxication, increase perceived wakefulness and control, delay sleep onset, and extend the duration of alcohol consumption (Andrade et al., 2023; Bonar et al., 2015) may operate similarly in gaming contexts by prolonging gaming sessions and reducing adolescents' awareness of problematic engagement. Similar to regular energy drinks, AmEDs have reported to be relatively easy for minors to obtain and conceal (Andrade et al., 2023).

Regarding cognitive functioning, research has found that adolescents with GD exhibit decreased episodic memory, attention, and problem-solving skills (Farchakh et al., 2020). Similarly, caffeine consumption in young adolescents has been associated with poorer episodic memory, working memory, cognitive flexibility, and processing speed (Zhang et al., 2020). Both GD and higher caffeine intake have also been associated with lower academic performance among adolescents, potentially due to negative effects on long-term focus and decision-making (Fontelles et al., 2025; Kim et al., 2017; Limone et al., 2023). Notably, two studies found that adolescents identified limiting or avoiding consumption of energy drinks as important factors for maintaining energy levels, focus, and academic performance, with one of these studies specifically examining high school students who participated in esports (Baumann et al., 2022; Ludden et al., 2017). Furthermore, a longitudinal study of adolescents reported a positive association between energy-drink consumption and gaming-related absenteeism from school and school-related stress one year later (Svensson et al., 2021). Social domains may also be impacted by GD (associated with impairments to social functioning and social isolation) and PCC (associated with mood swings and irritability that may strain relationships) in adolescents (Paulus et al., 2018; Ruiz and Scherr, 2019).

## 4 Public health framework and conclusions

Despite the link between GD/gaming and PCC/caffeine use in adolescents, research specifically examining the intersection of these behaviors appears limited. However, this narrative review highlights the multifaceted nature of GD/gaming and PCC/caffeine use in adolescents, considering multiple factors that warrant additional research and possible intervention, such as shared neurocognitive mechanisms, marketing influences, regulatory approaches, peer and parental dynamics, health and marketing literacy, sex-specific patterns, and overlapping negative consequences.

**Figure 1:**
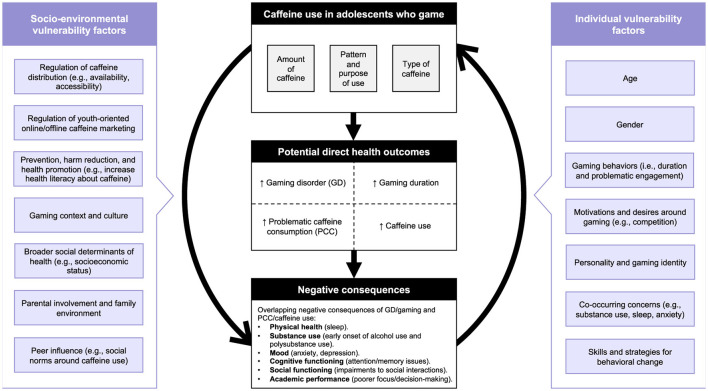
A public health framework addressing gaming-related caffeine use and associated health measures in adolescents.

Using findings from the present review, we propose the first public health framework of GD/gaming and PCC/caffeine use to lay the foundations for guiding future research, policy, and interventions to promote and protect the health of adolescents who consume caffeine and game (Figure 1). This conceptual model incorporates both socio-environmental and individual factors that contribute to adolescents' vulnerability to gaming-related caffeine use and its associated harms, including sleep difficulties.

This framework illustrates an indirect cyclic relationship involving caffeine use, gaming behaviors, and associated negative health concerns. Specifically, caffeine consumption may contribute to increased gaming duration and problematic gaming patterns (like GD), which may subsequently lead to negative outcomes such as sleep disturbances, mood dysregulation, and impaired academic performance. These concerns, in turn, could further reinforce adolescents‘ reliance on caffeine, perpetuating a worsening behavioral cycle. It is also important to recognize that socio-environmental and individual vulnerability factors may increase adolescents' susceptibility to initiate, escalate, and maintain involvement in these potentially mutually reinforcing behaviors. Therefore, simultaneously addressing both contextual vulnerabilities and the proposed core behavioral cycle may be essential to designing effective prevention and intervention strategies.

A deeper understanding of such interactions, guided by research and informed by adolescents' perspectives, has the potential to influence policy/regulation responses and public health approaches for prevention, harm reduction, and health promotion (i.e., upstream interventions) to mitigate the negative consequences of gaming-related use of caffeine (Mytton et al., 2024). At the downstream level, the model could inform the development of treatment guidelines for problematic gaming and targeted behavior change strategies that account for the associations between GD/gaming and PCC/caffeine use. This model is adapted from and expands on an existing framework on alcohol consumption and health outcomes (Rehm et al., 2010; Shield et al., 2014) by incorporating gaming- and caffeine-specific vulnerability factors. Additionally, it introduces a new component that addresses negative consequences that are shared across GD and PCC. As such, the proposed model presents an opportunity to encourage further research in comorbidity and multimorbidity, which are health issues that have frequently challenged healthcare professionals, researchers, and policymakers (Chudasama et al., 2021; Park et al., 2023, 2021; Rokkum and Gentile, 2018).
